# Examining PI3K-signaling-dependent regulation of lens organelle free zone formation via immunolocalization and immunoblotting in chick embryos

**DOI:** 10.1016/j.xpro.2023.102569

**Published:** 2023-09-14

**Authors:** Rifah Gheyas, A. Sue Menko

**Affiliations:** 1 Department of Pathology and Genomic Medicine, Thomas Jefferson University, Philadelphia, PA 19107, USA

**Keywords:** Cell Biology, Developmental Biology, Microscopy

## Abstract

The elimination of lens organelles during development, required for mature lens function, is an autophagy-dependent mechanism induced through suppression of PI3K signaling. Here, we present a protocol for investigating the signaling pathways responsible for induction of the formation of this lens organelle free zone. We describe steps for preparation of lens organ culture and use of signaling pathway inhibitors. We then detail procedures for analyzing their impact using both confocal microscopy imaging of immunolabeled lens cryosections and immunoblot approaches.

For complete details on the use and execution of this protocol, please refer to Gheyas et al. (2022).^1^

## Highlights

• Steps are described for preparing lens organ cultures
• Instructions for treating organ cultures with signaling pathway inhibitors
• Technique for revealing lens organelle spatial distribution using immunolocalization
• Determine molecular changes in *in organelle* proteins through immunoblotting

## Abstract (editor highlights)

Publisher’s note: Undertaking any experimental protocol requires adherence to local institutional guidelines for laboratory safety and ethics.

## Graphical abstract

**Figure:**
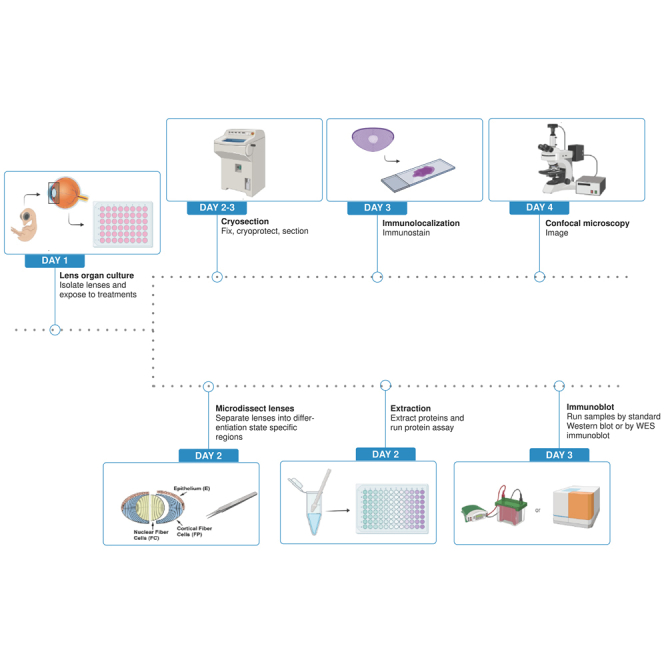

## Before you begin

Fertilized white leghorn chicken eggs should be acquired from sources such as Poultry Futures (Lilitz, PA) or Charles River (Wilmington, MA) and incubated in a GQF Digital Sportsman Cabinet (egg incubator) which is set to 37°C and humidity between 45%-55% until use. Determine the embryonic stage of interest to prepare the chick embryo lens organ cultures. These cultures can be grown successfully from E8-E15, depending on the particular time of development at which the investigator wishes to examine the impact of inhibiting a signaling pathway. Inhibitors should be purchased and reconstituted prior to beginning this protocol. Antibodies which will be the targets of interest for analysis using the immunolocalization/immunoblotting protocols should be determined, as well as the appropriate antibody dilution. While this protocol is focused on understanding the role of PI3K signaling pathways in the regulation of lens organelle loss, we have used that same approach to examine the role of other pathways.

### Institutional permission

The protocol uses lenses isolated from chick embryos that were performed in compliance with the Association for Research in Vision and Ophthalmology Statement for the Use of Animals in Ophthalmic and Vision Research. Approval for performing such studies must be obtained from the relevant site’s Institutional Animal Care and Use Committee (IACUC).

## Key resources table

**Table:** 

REAGENT or RESOURCE	SOURCE	IDENTIFIER
**Antibodies**		

TOM20 (1:100)	Cell Signaling Technologies	Cat# 42406; RRID:AB_2687663
TOM20 (1:50)	Santa Cruz	Cat# sc-17764; RRID:AB_628381
Calreticulin (1:50)	Santa Cruz	Cat# sc-166837; RRID:AB_2069628
pAkt ser473 (1:50)	Cell Signaling Technologies	Cat# 4060; RRID:AB_2315049
pAkt thr308 (1:50)	Cell Signaling Technologies	Cat# 2965; RRID:AB_2255933
Akt (1:100)	Cell Signaling Technologies	Cat# 9272; RRID:AB_329827
p-p70S6K (1:50)	Cell Signaling Technologies	Cat# 97596; RRID:AB_2800283
p70S6K (1:50)	Cell Signaling Technologies	Cat# 2708; RRID:AB_390722
LAMP1 (1:50)	Abcam	Cat# ab24170; RRID:AB_775978
Rhodamine (TRITC)-conjugated Goat Anti-Mouse IgG (1:100)	Jackson ImmunoResearch	Cat# 115-025-003; RRID:AB_2338478
Alexa Fluor 488-conjugated AffiniPure Goat Anti-Rabbit IgG (1:100)	Jackson ImmunoResearch	Cat# 111-545-144; RRID:AB_2338052

**Chemicals, peptides, and recombinant proteins**		

LY294002	Selleckchem	S1105
CH5132799	Selleckchem	S2699
MK-2206	Selleckchem	S1078
Dimethyl sulfoxide (DMSO)	Sigma Aldrich	D5879
Triton-X 100	Fisher Scientific	9002-93-1
Tween-20	Fisher Scientific	BP337-500
BSA	Fisher Scientific	BP9706
Paraformaldehyde (16%)	Electron Microscopy Sciences	15710-S
Goat serum	Meridian Life Science, Inc	N66001G
Ethanol	Decon Labs	2701
Dulbecco’s phosphate-buffered salt solution (DPBS)	Corning	MT21030CV
DAPI	BioLegend	422801
Fetal bovine serum	Gibco	A31605-01
Medium 199	Gibco	11150-059
L-Glutamine	Corning	25-005-Cl
Penicillin-streptomycin	Corning	30-002-Cl
ProLong Diamond Antifade	Thermo Scientific	P36970
Pierce ECL plus western blotting substrate	Thermo Scientific	32132
Goat anti-rabbit IgG HRP conjugate	Bio-Rad	1706515
Goat anti-mouse IgG HRP conjugate	Bio-Rad	1706516
Protease/Phosphatase Inhibitor Cocktail	Cell Signaling Technologies	5872
Halt Phosphatase Inhibitor Cocktail	Thermo Fisher Scientific	78420
NaCl	Fisher Scientific	S271-3
Na_2_HPO_4_	Fisher Scientific	S374-500
Dextrose	Fisher Scientific	D16-500
Tris base	Fisher Scientific	BP152-1
Imidazole	Sigma	I0125
MgCl_2_	Fisher Scientific	M33-500
EDTA	Sigma	E5134
n-Octylglucoside	Sigma	29836-26-8
Glycine	Fisher Scientific	BP-381-1
SDS	Fisher Scientific	BP1311-1
Glycerol	Fisher Scientific	BP229-1
2-Mercaptoethanol	Fisher Scientific	03446
Bromophenol blue	Sigma	B0126
Sucrose	Fisher Scientific	S5-3

**Critical commercial assays**		

BCA assay	Thermo Fisher Scientific	23250

**Experimental models: Organisms/strains**		

Embryonic chicken		

**Software and algorithms**		

Compass for Simple Western	Bio-Techne	https://www.bio-techne.com/resources/instrument-software-download-center
Zen blue	Zeiss	https://www.zeiss.com/microscopy/en/products/software/zeiss-zen-lite.html
GraphPad PRISM	GraphPad	https://www.graphpad.com/features
Softmax Pro	Molecular Devices	https://www.moleculardevices.com/products/microplate-readers/acquisition-and-analysis-software/softmax-pro-software
AlphaView SA	Bio-Techne	https://alphaview-sa1.software.informer.com/

**Other**		

Cryostat	Thermo Fisher Scientific	Microm HM 500
Zeiss Confocal Microscope	Zeiss	LSM800
WES	Bio-Techne	
FluorChem E&M Imager	Bio-Techne	
Dumont Standard Forceps	Fine Science Tools	11251-20 and 11251-21
PolyFreeze (tissue freezing medium)	Polysciences, Inc	25115-1
Square shape mold 22 × 22 mm	Electron Microscopy Sciences	70182
12–230 kDa Separation Module	Bio-Techne	SM-W001
Anti-Rabbit Detection	Bio-Techne	DM-001
Anti-Mouse Detection	Bio-Techne	DM-002
EZ Standard Pack	Bio-Techne	PS-ST01EZ-8
Novex Tris-Glycine Gels 4–12%	Thermo Fisher	XP04120BOX
Novex Tris-Glycine Gels 8–16%	Thermo Fisher	XP08160BOX
Hoefer Power Supplies	Fisher Scientific	PS300-B
Hoefer TE22 Mini Tank Blotting Unit	Fisher Scientific	03-500-216
XCell SureLock Mini-Cell Electrophoresis	Thermo Fisher	EI0001
ImmEdge Pen	Vector Laboratories	H-4000
Immobilon-P transfer membranes PVDF	Millipore	IPVH00010
Chromatography paper	GE HealthCare Life Sciences	3030-681
Petri dish, stackable	Fisher Scientific	FB0875711Z
48-Well cell culture plate	Corning	3548
Superfrost Plus Microscope Slides	Fisher Scientific	22037246
Microscope cover glass	Fisher Scientific	12542B
Pellet pestles	Fisher Scientific	12-141-363
Isotemp heat block	Fisher Scientific	88860022
Centrifuge 5417R	Eppendorf	540716066
Sorvall Legend X1R Centrifuge	Thermo Scientific	75004261
SpectraMax 340PC	Molecular Devices	
GQF Digital Sportsman Cabinet	Incubator Warehouse	1502

## Materials and equipment

**Table:** Tris-Dextrose Buffer

Reagent	Final concentration	Amount
NaCl	137 mM	8 g
Na_2_HPO_4_	704 μM	0.1 g
Dextrose	5.50 mM	1 g
Tris Base	8 mM	1 g
milliQ H_2_O	N/A	Up to 1 L
**Total**	N/A	**1 L**

Note on storage conditions: pH 7.4, autoclaved, stored at ∼22°C for up to 6 months.

**Table:** Imidazole Buffer

Reagent	Final concentration	Amount
Imidazole	10 mM	0.68 g
NaCl	100 mM	5.84 g
MgCl_2_	2 mM	0.2 g
EDTA	6 mM	1.86 g
milliQ H_2_O	N/A	Up to 1 L
**Total**	N/A	**1 L**

Note on storage conditions: pH 7.4, long term storage at -20°C up to 3 years, short term storage at 4C.

**Table:** 2X OG/T

Reagent	Final concentration	Amount
n-Octylglucoside	90 mM	26.32 g
Triton-X100	2%	20 mL
Imidazole	10 mM	0.68 g
NaCl	100 mM	5.84 g
MgCl_2_	2 mM	0.2 g
EDTA	6 mM	1.86 g
milliQ H_2_O	N/A	Up to 1 L
**Total**	**N/A**	**1 L**

Note on storage conditions: long term storage at -20°C up to 3 years.

**Table:** 10x Running Buffer

Reagent	Final concentration	Amount
Tris Base	240 mM	29.08 g
Glycine	2 M	144 g
SDS	1%	10 mL
milliQ H_2_O	N/A	Up to 1 L
**Total**	**N/A**	**1 L**

Note on storage conditions: pH 8.3, stored at ∼22°C for up to 6 months.

**Table:** 10X Transfer Buffer

Reagent	Final concentration	Amount
Tris Base	250 mM	30.3 g
Glycine	2 M	144 g
milliQ H_2_O	N/A	Up to 1 L
**Total**	**N/A**	**1 L**

Note on storage conditions: pH 8.3, stored at 4°C for up to 6 months.

**Table:** 10X TBS

Reagent	Final concentration	Amount
Tris Base	200 mM	24.23 g
NaCl	1.5 M	80.06 g
milliQ H_2_O	N/A	Up to 1 L
**Total**	**N/A**	**1 L**

Note on storage conditions: pH 7.6, stored at ∼22°C for up to 1 year.

**Table:** 1x TBS-T

Reagent	Final concentration	Amount
10x TBS	N/A	100 mL
Tween-20	0.1%	1 mL
milliQ H_2_O	N/A	900 mL
**Total**	**N/A**	**1 L**

Note on storage conditions: stored at ∼22°C for up to 6 months.

**Table:** 2X Sample Buffer

Reagent	Final concentration	Amount
SDS	4%	4 g
Glycerol	20%	20 mL
2-ME (add fresh)	2%	2 mL
Bromophenol blue	0.01%	0.001 g
4X Tris Cl/SDS	N/A	25 mL
milliQ H_2_O	N/A	Up to 100 mL
**Total**	**N/A**	**100 mL**

Note on storage conditions: stored at -80°C for up to 2 years.

**Table:** 4x Tris Cl/SDS

Reagent	Final concentration	Amount
Tris Base	N/A	6.05 g
SDS	0.4%	0.4 g
milliQ H_2_O	N/A	Up to 100 mL
**Total**	**N/A**	**100 mL**

Note on storage conditions: pH 6.8, filter, store at 4°C for 1 month.

**Table:** Complete Media

Reagent	Final concentration	Amount
Medium 199	N/A	88 mL
Fetal bovine serum	10%	10 mL
L-glutamine	1%	1 mL
Penicillin-Streptomycin	1%	1 mL
**Total**	**N/A**	**100 mL**

Note on storage conditions: stored at 4°C for up to 1 week.

**Table:** Permeabilization Buffer

Reagent	Final concentration	Amount
Triton X-100	0.5%	50 μL
PBS	N/A	10 mL
**Total**	**N/A**	**10 mL**

Note on storage conditions: stored at 4°C for up to 1 week.

**Table:** Block Buffer

Reagent	Final concentration	Amount
goat serum	5%	0.5 mL
BSA	1%	0.1 g
PBS	N/A	Up to 10 mL
**Total**	**N/A**	**10 mL**

Note on storage conditions: stored at 4°C for up to 1 week.

**Table:** 30% Sucrose

Reagent	Final concentration	Amount
Sucrose	30%	15 g
PBS	N/A	Up to 50 mL
**Total**	**N/A**	**50 mL**

Note on storage conditions: stored at 4°C.

## Step-by-step method details

### DAY 1 – preparing lens organ cultures

**Timing: ∼1 h per 30 eggs**

This section describes the preparation of lens organ cultures isolated from chick embryos, in which lenses continue to undergo normal development as occurs *in vivo*, including the elimination of organelles to form the lens Organelle Free Zone.

**Figure 1:**
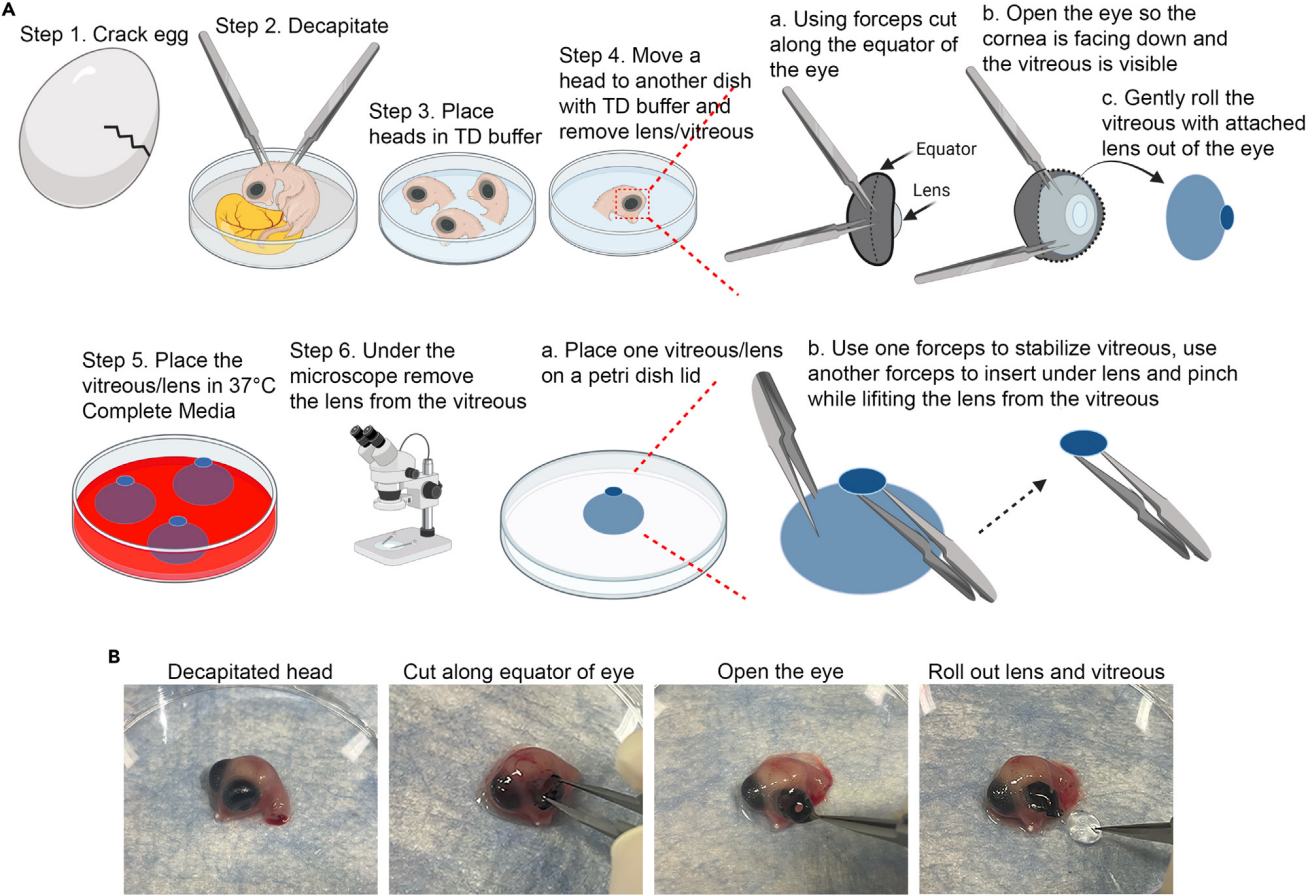
Isolating embryonic chick lenses (A) Eggs are cracked one at a time into a 100mm Petri dish where the embryos are decapitated, an incision is made along the equator of the eye to allow the lens/vitreous to roll out with the least amount of damage to the lens, the lenses are then pinched off from the vitreous and placed into well plates with Complete Medium to create lens organ cultures. (B) First the head is removed, after which an incision is made in the eye to create an opening from which the lens together with its associated vitreous is rolled out (corresponding to Steps A3-4).
  c. Place the heads in a separate dish filled with Tris-Dextrose buffer (in Materials and equipment) (Figure 1, step 3).
  d. Move one head at a time to another dish filled with Tris-Dextrose buffer. Use standard forceps to cut open the eye along the equator to create an opening large enough to roll out the lens together with its attached vitreous humor (Figure 1, step 4a-b).
  e. Remove the lens/vitreous by grabbing the vitreous with standard forceps (Figure 1, step 4b-c) and placing it in the last dish which is filled with Complete Medium (in Materials and equipment) prewarmed to 37°C (Figure 1, step 5).
2. Under the microscope, place one lens/vitreous on a petri dish lid (Figure 1, step 6a).
3. Use forceps to stabilize the vitreous while using another set of forceps to pinch underneath the lens to detach the lens from the vitreous (Figure 1, step 6). The lenses will be used in lens organ cultures.
4. Lens organ cultures
  a. After removing the lens from the vitreous, immediately transfer each isolated lens to a 48 well plate filled with 0.5 mL Complete Medium per well. Place each lens in a separate well.
  b. Once the lenses are added to the 48 well plate, place the plate in a 37°C humidified tissue culture incubator with 5% CO_2_ and normoxia for 1 h.
  c. After 1 h of incubation, remove any lenses that develop opacities from the study prior to treatments.

***Note:*** This lens organ culture protocol is a modification of one that was previously published.^2^^,^^3^

***Note:*** The frequency of lenses that develop opacities will depend on how gently the lenses are handled as any damage by the forceps will result in opacities. One should practice making lens organ cultures to understand how many eggs/lenses one needs to have enough transparent lenses for an experiment.

1. Isolate embryonic chicken lenses (E12) – about 5 lenses/condition for immunolocalization studies and 5–10 lenses/condition for immunoblot studies – perform all steps under sterile conditions.
  a. Place 4 100 mm petri dishes in a laminar flow hood.
  b. Use one dish to crack eggs and decapitate embryos using sharp Dumont Standard Tip Forceps (Figure 1, steps 1 and 2).

### DAY 1 – Use of signaling pathway inhibitors in lens organ cultures

**Timing: 30 min**

This section provides the protocol for treating chick embryo lenses in organ culture to signaling pathway inhibitors for the purpose of evaluating the role of the targeted pathways in the differentiation of lens fiber cells and the development of the lens.

5. Prepare the inhibitors which will be used to treat the lens organ cultures.
  a. Dilute inhibitors from a stock solution of 10 mM in 37°C pre-warmed Complete Medium.
  b. Use the vehicle of the inhibitors, DMSO, as control. The largest volume of inhibitor used in the experiment is that of the vehicle DMSO, which is diluted using Complete Medium prewarmed to 37°C prior to adding to the control lenses.
  c. Once the inhibitors are added to the media, place the well plate in a 37°C humidified tissue culture incubator with 5% CO_2_ for 24 h.
  d. Determine the efficacy of inhibitors to block a signaling pathway by examining impact on downstream signaling effectors using an immunoblot approach.

***Note:*** To study PI3K inhibition, the inhibitors we used included the pan-PI3K inhibitors LY294002 (100 μM, Selleckchem) or CH5132799 (25 μM or 100 μM, Selleckchem) or the Akt-specific inhibitor MK-2206 (10 μM, Selleckchem). Efficacy of PI3K inhibitors to block PI3K signaling pathways was determined based on inhibition of Akt activity using an immunoblot approach.

### DAY 2 – Immunolocalization protocol

**Timing: 26 h**

**Timing: 2 h (for step 6)**

**Timing: 24 h (for step 7)**

This section describes the first steps in preparing lenses for immunolocalization analysis that includes protein fixation and cryoprotection of the lenses, so that tissue integrity is preserved through the next steps.

6. Fixation
  a. Wash lenses twice with PBS (in the 48 well plate) and then fix with 0.5 mL/well 4% paraformaldehyde (16% paraformaldehyde stock solution diluted in PBS) for 2 h at 4°C.
7. Cryoprotection
  a. Wash lenses twice with PBS and then cryoprotect in 0.5 mL/well 30% sucrose (in PBS) for 24 h at 4°C.

***Note:*** May store lenses in sucrose for 1 month.

### DAY 2 – Immunoblotting protocol

**Timing: ∼4 hrs**

**Timing: ∼1 min per lens (for step 8)**

**Timing: 2 h (for step 9)**

**Timing: 1 h (for step 10)**

This section describes the protocol for preparing the treated lenses for an analysis of protein expression that will use a biochemical approach. Since we are examining the impact of signaling pathway inhibitors on features of lens differentiation, the first step described here is to separate the three primary regions of differentiation by microdissection, followed by how to extract the protein and determine the protein concentration in each sample.

**Figure 2:**
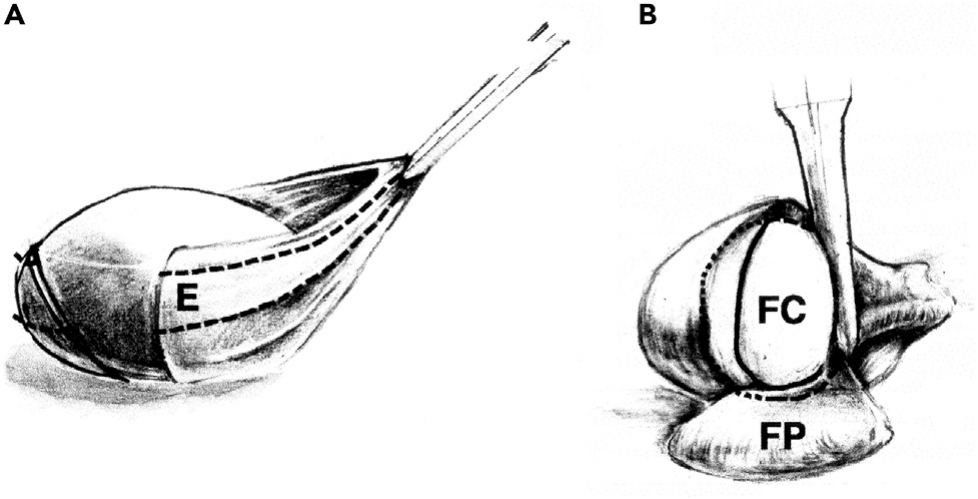
Microdissection of lenses into three differentiation state specific regions (A and B) Under a dissecting microscope, forceps are used to create a shallow puncture in the anterior epithelium, denoted by (E), located at the top of the lens. The tear in the epithelium is widened and the epithelium is grabbed along the tear by the tips of the forceps following which the entire epithelium pulled away from the fiber cell mass as seen in (A). Forceps are then used to remove the cortical fiber cells (FP) from the central fiber cells (FC), shown in (B). This process is relatively straightforward as the cells in the FC form a tightly packed mass in the center of the lens, while the FP consists of a looser aggregate of cells. Each microdissected region is then placed/stored in Imidazole Buffer until it is time to perform protein extraction.
    ii. Repeat the microdissection procedure for each lens in the study and place the individual tissue fractions on ice in separate 0.5 mL microfuge tubes containing 1-2 μL Imidazole Buffer per lens dissected (in Materials and equipment) with protease/phosphatase inhibitors to protect against serine/threonine/tyrosine phosphatases (in key resources table) at 1:100 dilution, combining all like fractions together in one tube.

***Note:*** At this point in the protocol samples can be stored at -80°C until ready to use.

***Note:*** The lens microdissection protocol is a modification of one that was previously published.^4^

8. Microdissection of lenses
  a. Using a dissecting microscope and standard forceps, microdissect each lens to yield three unique differentiation state specific fractions.
    i. Place a lens on the lid of a 100 mm petri dish. Remove the lens epithelium first, then separate the cortical fiber cells from the central, nuclear fiber cells (Figure 2).

9. Protein extraction
  a. Add the extraction buffer, 2X OG/T (in Materials Table), to each sample at an equal volume to the Imidazole Buffer.
  b. Homogenize the tissue using disposable plastic pestles that fit the microfuge tube; continue homogenizing until tissue pieces are no longer visible (approximately 30 s).
  c. Incubate the homogenized tissue on ice for 15 min.
  d. Centrifuge the extracted samples for 5 min at 15000 x g.
  e. Remove the supernatant carefully and place in new microfuge tubes.

***Note:*** May store extracted samples at -80°C until ready to use.

10. BCA Protein Assay
  a. Dilute samples in Imidazole Buffer (in Materials Table).
    i. Dilute epithelial cell samples obtained from 5-10 lenses as above at 1:20 for the protein assay.
    ii. Dilute fiber cell samples obtained from 5-10 lenses as above at 1:80.
  b. Generate a standard curve (0.025 mg/mL to 2 mg/mL) using Bovine Serum Albumin protein standards. Load 5 μL in triplicate in a 96 well plate.
  c. Load each sample at 5 μL in triplicate.
  d. Load a blank sample at 5 μL in triplicate (Imidazole Buffer).
  e. Mix reagents A and B in a 50:1 ratio and add 200 μL of this solution to each well.
  f. Cover and incubate the plate for 30 min at 37°C.
  g. Measure the absorbance at 562nm on a SpectraMax 360 plate reader.

***Note:*** The protein extracted from 5-10 lenses is about 10 μg for epithelial cell fractions and 15 μg each for cortical fiber and nuclear fiber cell fractions.

***Note:*** May store samples at -80°C until ready to use.

### DAY 3 – Immunolocalization protocol

**Timing: ∼2 days**

**Timing: ∼2 min per lens (for step 11)**

**Timing: 30 min**–**1 h per lens (for step 12)**

**Timing: 1–2 days (for step 13)**

This section shows how to properly freeze the cryoprotected lenses, section them using a cryostat, and then provides a detailed guide to the immunolabeling of lens sections.

11. Freezing lenses
  a. Remove lenses from the sucrose cryoprotectant using forceps and embed in about 4 mL tissue freezing media in a (22 × 22mm) square mold (Figure 3).

**Figure 3:**
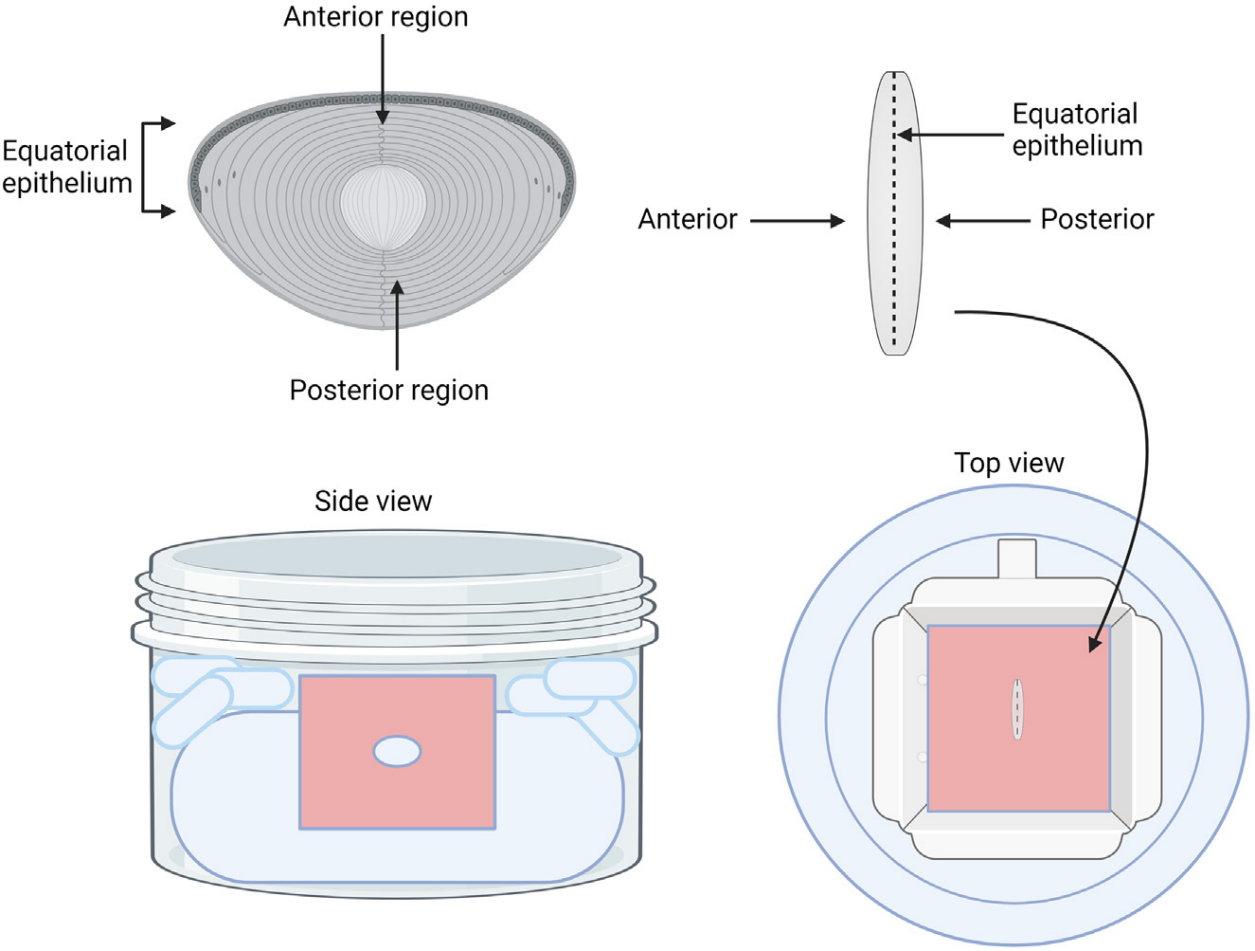
Embedding/freezing lenses in tissue freezing media to allow for cryosectioning Lenses are positioned within the tissue freezing media so that the equatorial epithelium is facing up and the anterior/posterior sides of the lens are facing to the right and left. When these lenses are cryosectioned, all of the different differentiation state specific regions of the lens are visible at once.
  b. Position lenses under a dissecting scope so that the equatorial region is facing up (Figure 3).
  c. Freeze lenses embedded in freezing media by placing the mold in a dry ice/ethanol bath (Figure 3).
12. Cryosectioning
  a. Section frozen blocks using a Microm HM 500 Cryostat, typically cutting 20 μm thick sections (Figure 4).

**Figure 4:**
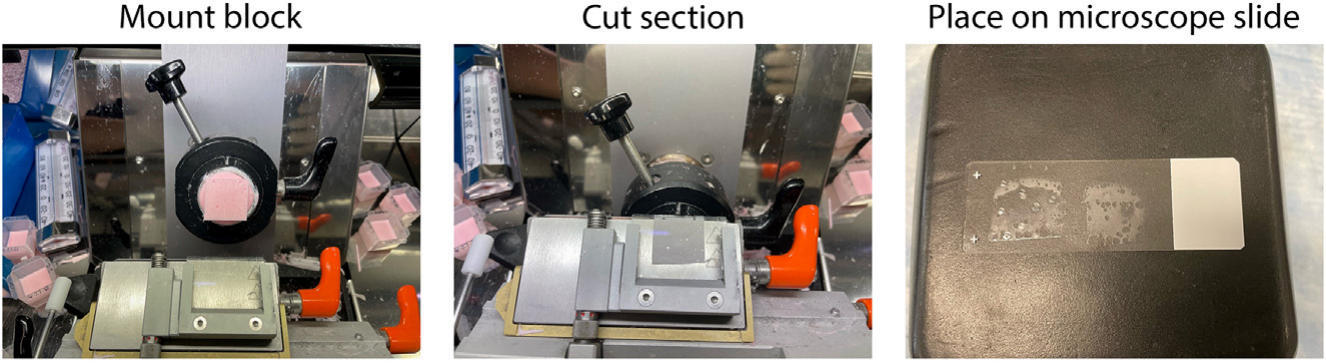
Cryosectioning frozen tissue Embedded lenses are mounted to the chuck and sections are cut. They are then transferred to microscope slides on which they can be immunostained.
  b. Pick up frozen sections on a microscope slide by touching the slide to the tissue (Figure 4).

***Note:*** Frozen blocks are stored at -80°C and cut sections are stored at -20°C.

***Note:*** Cryostat is set to -25°C. Blocks are placed inside for a minimum of 20 minutes prior to sectioning to allow tissue to acclimate.

***Note:*** Cryosections can be cut at 6–20 μm.

13. Immunostaining
  a. Choose sections that were cut through the center of the lens.
  b. Using a hydrophobic pen, draw a square around each section.
  c. Add 100 μL Permeabilization Buffer (in Materials Table) to the square created with the hydrophobic pen. Incubate for 30 min at ∼22°C. This step will remove traces of freezing media before staining.
  d. Remove the Permeabilization Buffer.
  e. Incubate the sections in 100 μL Block Buffer (in Materials Table) at ∼22°C for 1 h.
  f. Remove Block Buffer.
  g. Incubate sections in primary antibodies diluted in Block Buffer at either 37°C for 3 h or 4°C for ∼16 h.
  h. Remove primary antibody and wash sections with PBS for 5 min 3 times.
  i. Incubate with appropriate secondary antibody diluted in the Block Buffer and containing DAPI (1:1000) at 37°C for 2 h.
  j. Remove secondary antibody and wash sections with PBS for 5 min 3 times.
  k. Add a drop of ProLong Diamond Antifade mounting media and cover with a Microscope Cover Glass coverslip.

***Note:*** Center sections are used exclusively as organelle loss is initiated in the center of the lens.

***Note:*** For secondary alone control studies it is not necessary to use centermost sections.

***Note:*** Acclimate the frozen sections to 22°C (about 10 minutes) before beginning the immunolocalization protocol.

***Note:*** It may be necessary to optimize incubation times for some primary antibodies.

### DAY 3 – Immunoblotting protocol

**Timing: ∼2 h**–**3 days depending on technique used**

**Timing: 2 h (for step 14)**

**Timing: 2–3 days (for step 15)**

In this section we describe two very different immunoblotting protocols that can be used to accomplish the goal of determining and quantifying protein expression, one using the WES Simple Western System from Bio-Techne and a more typical Western Blot approach. The WES immunoblot provides a fast, highly reproducible method of immunoblotting in which all samples, antibodies, and reagents are loaded into a plate and proteins are separated by capillary electrophoresis. The WES system also quantifies the results. For Western blotting, the protocol described here includes the steps involved in gel electrophoresis, transfer to a PDVF membrane, and the exposure of the membrane to the primary antibody.

**Figure 5:**
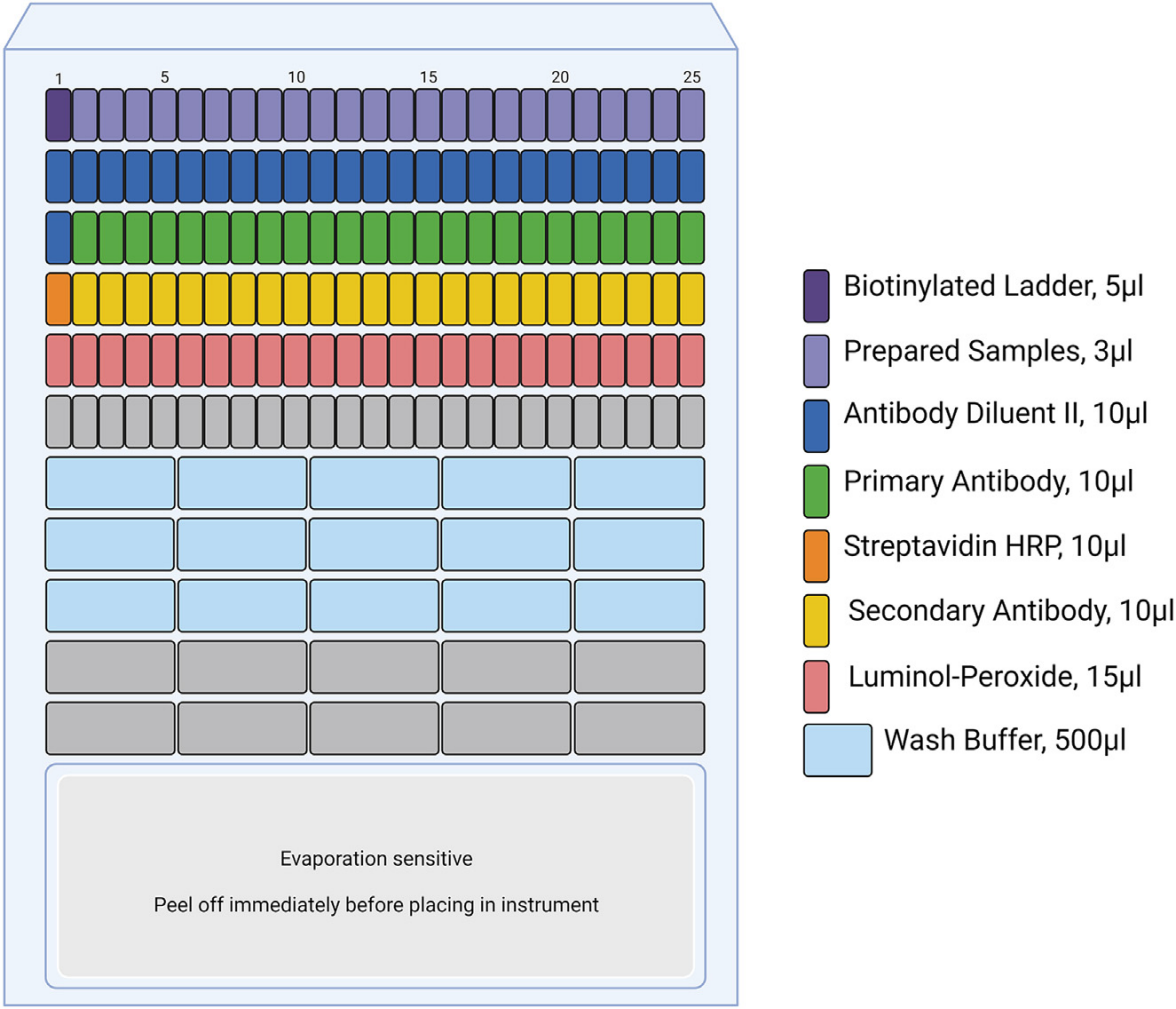
Loading of WES plate Each reagent is loaded into specific wells at specific volumes within the WES plate. The plate is centrifuged, the seal covering the bottom of the plate is removed, and the plate is placed into the WES machine with a set of capillaries prior to running.
  d. Load antibody Diluent II (provided with the 12–230 kDa Separation Module) at 10 μL /well into the second row of the plate (Figure 5).
  e. For the ladder, load Antibody Diluent II (provided with the 12–230 kDa Separation Module) into the first well of the third row (Figure 5).
  f. Dilute primary antibodies in Antibody Diluent II to an optimized concentration. Load 10 μL/well across the third row (Figure 5).
  g. For the ladder, load 10 μL Streptavidin HRP (provided with the Anti-Rabbit/Mouse Detection kit) into the first well of the fourth row (Figure 5).
  h. Load 10 μL of secondary antibody (provided with the Anti-Rabbit/Mouse Detection kits) to the remainder of the wells across the fourth row (Figure 5).
  i. Mix luminol and peroxide (provided with the Anti-Rabbit/Mouse Detection kit) in a 1:1 ratio and add to all the wells in the fifth row at 15 μL /well (Figure 5).
  j. Cover the plate with the supplied lid and centrifuge at 1000 x g for 5 min.
  k. Load 500 μL of wash buffer (provided with the 12–230 kDa Separation Module) in all the wells of the seventh, eighth, and ninth rows (these are the first 3 rows of larger wells) (Figure 5).
  l. Remove the seal from the lower part of the plate, just prior to running.
  m. Load the plate and a set of capillaries (part of 12–230 kDa Separation Module) into the WES machine.
  n. Using Bio-Techne’s Compass software, label a plate template with the contents of each well.

***Note:*** Plate will run for about 3 hrs.

***Note:*** The optimal protein concentration for each sample is between 0.005-3 μg.

14. WES Immunoblot
  a. Prepare samples to be loaded and run. For each well that will be run on a WES, the preparation must include your sample, 0.6 μL of Fluorescent Master Mix (provided with the manufacturer’s EZ Standard Pack), and bring your sample to a final volume of 3 μL with sample buffer (diluted to 0.1X from 10X sample buffer stock provided with the manufacturer’s Separation Module). While the ratios of these reagents will stay the same, the final volumes should reflect the number of wells that will be loaded with the sample.
  b. Heat the prepared samples (as above) at 100°C for 5 min.
  c. Load 5 μL of a biotinylated ladder (included with the EZ Standard Pack) into the first well of the first row. Then load the prepared samples into the rest of the first row of wells of the plate, 3 μL /well (provided in the 12–230 kDa Separation Module). There are 24 wells available for sample loading (Figure 5).

https://resources.bio-techne.com/bio-techne-assets/docs/software/Simple%20Western/Wes/Wes%20User%20Guide_Rev%208.pdf.

15. Western blot
  a. Polyacrylamide gel electrophoresis (3 h)
    i. Prepare samples for electrophoresis using Novex Tris-Glycine 10 well gels. The volume per well should not exceed 40 μL after adding an equal volume of 2X Sample Buffer (formulation found in Materials Table). Optimal protein concentration for lens fiber cell lysates is approximately 50 μg/well. While the ratios of these reagents will stay the same, the final volumes should reflect the number of wells that will be loaded with the sample.
    ii. After adding 2x Sample Buffer at a 1:1 ratio, heat samples for 10 min at 100°C.
    iii. Choose pre-made polyacrylamide gradient gels of either 4%–12% or 8%–16% (10 wells/gel) depending on the molecular weights of the proteins to be examined.
    iv. Wash the wells in each lane 3 times with 1x Running buffer (in Materials Table), leaving running buffer in at the last wash step.
    v. Place gel in an XCell SureLock Mini-Cell Electrophoresis apparatus. This apparatus is able to run two gels simultaneously, if only running one gel, use a place holder in the position of second gel. Fill middle of apparatus with 1x Running buffer.
    vi. Connect to power source and run at 80 V until bands run through stacking gel, then increase up to 120 V.
    vii. Stop when the dye front reaches the bottom of the gel.
  b. Transferring the protein bands from the gel to a PVDF membrane (4 h)
    i. Cut the PVDF membrane to the size of the gel (8 cm × 8 cm).
    ii. In a small tray, hydrate the PVDF membrane in 100% methanol for 10 s and then wash with milliQ water.
    iii. In a larger tray, add 1x Transfer buffer (in Materials Table) and place it in an opened gel holder cassette from a Hoefer TE22 Mini Tank Blotting Unit with its black side down. First, place a foam sponge on the cassette, then a piece of chromatography paper, followed by the polyacrylamide gel, the PVDF membrane, another piece of chromatography paper, and another sponge. Close the cassette.
    iv. Place loaded cassettes in transfer apparatus.
    v. Fill the apparatus between min and max lines with 1x Transfer buffer.
    vi. Place the lid on the apparatus, place it in a 4°C glass front refrigerator, connect to a power source and start the transfer (run at 280 mA for 210 min) for membranes with a pore size of 0.45 μm.
      ***Note:*** We have run target proteins between 20 kDa and 280 kDa at 280 mA for 210 minutes.
  c. Immunoblotting (primary antibody incubated ∼16 h)
    i. Blocking step: Remove the PVDF membrane from cassette and place in 5% milk (10 g of powdered milk to 200 mL of 1X TBS-T (in Materials Table), pH to 7.6, and filter before use) in a tray for 1 h at 22°C on a gentle rocking apparatus.
    ii. Pour out the 5% milk solution and add primary antibodies diluted in 5% milk; rock ∼16 h at 4°C.
      ***Note:*** Position cassette so that the black side faces the back of the transfer apparatus and the gel transfers to the membrane.
      ***Note:*** Certain primary antibodies need to be diluted in BSA.

### DAY 4 – Immunolocalization protocol

**Timing: 30 min**–**1 h/section**

This section describes how confocal microscopy imaging is used to examine the impact on the spatiotemporal localization of the molecules of interest after exposing lenses in organ culture to signaling pathway inhibitors.

16. Confocal microscopy
  a. Using an LSM800 Zeiss Confocal Microscope, identify the lens section under low magnification (5X or 10X) and then switch to 40X magnification.
  b. Determine the optical section thickness for a specific study depending on the area of interest. If the entire thickness of the section is needed, then a z-stack of the tile should be acquired.
  c. Using the most brightly stained experimental condition, go through each channel and adjust the laser wavelength and detector gain (up to 800V) until the staining intensity is bright/clear without over saturating. If the range indicator shows that the staining is over saturated, decrease the laser wavelength.
  d. Before acquiring the image, use the tile application in Zeiss’ confocal software (Zen), set up a tile that extends across the entire lens section. As lenses increase in size during development the number of tiles needed to acquire this image will depend on lens size.
  e. After images have been acquired, use Zen software to adjust brightness/contrast, overlay specific channels, measure staining intensity, and create orthogonal projections, among other options.

***Note:*** For our studies, we adjust the settings to acquire an image using an optical section thickness of 0.7 μm.

***Note:*** Acquiring a 10 × 10 tile using three different channels at a scan speed of 7 takes about 20 minutes.

***Note:*** If the tissue section is not flat, add support points where needed and the software will provide a cohesive and smooth image.

***Note:*** While we are using an LSM800 Zeiss Confocal Microscope, any microscope and its accompanying software may be used. A confocal microscope is able to image a single optical plane but other types of microscopes can be used to image immunolabeled tissue sections.

### DAY 4 – Immunoblotting protocol

**Timing: 3–4 h**

Here is described the final steps of the standard immunoblotting protocol that allows visualization and quantification of the proteins of interest.

17. Western blot (continued)
  a. Wash the PVDF membrane with TBS-T for 30 min at 22°C.
  b. Add secondary antibody tagged with HRP diluted in 5% milk and incubate for 2 h at 22°C.
  c. Wash the membrane with TBS-T for 30 min at 22°C.
  d. Incubate the membrane in ECL plus for 5 min.
  e. Detect bands using a FluorChem E&M imager.

## Expected outcomes

### Use of immunoblot analysis to examine outcomes of the exposure of lenses in organ culture to signaling pathway inhibitors on the induction of autophagy and the premature elimination of lens organelles

**Figure 6:**
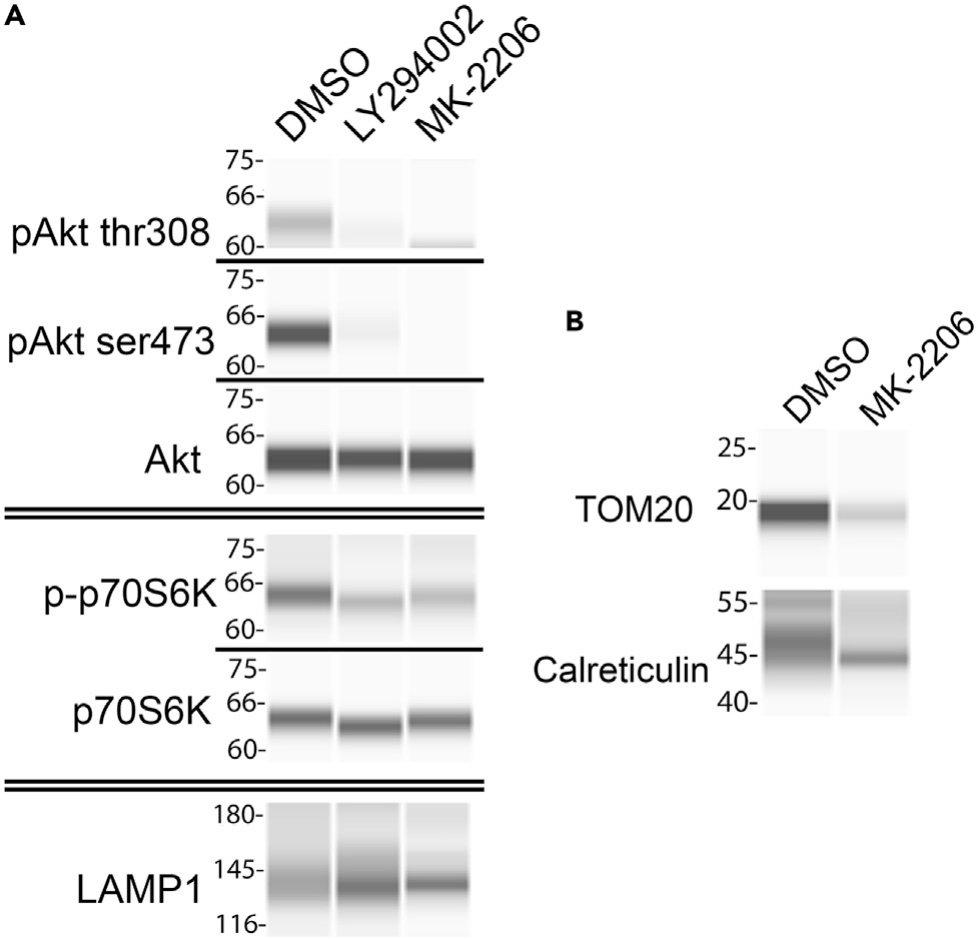
Suppression of PI3K/Akt signaling leads to the induction of autophagy and the removal of non-nuclear organelles (A and B) Phosphorylation of Akt is inhibited by LY294002 and MK-2206, leading to the suppression of p70S6K activation and induction of autophagy as shown by the increased expression of the lysosomal marker LAMP1 (A). Inhibition of Akt by MK-2206 also results in the loss of mitochondria as shown by TOM20 and endoplasmic reticulum (ER) as shown by immunoblot for calreticulin (B).

Small molecule inhibitors that specifically target distinct elements of signaling pathways can be used effectively in lens organ cultures to link the pathway to function in the lens differentiation process. The efficacy of inhibitors to block the targeted signal is easily determined using an immunoblot approach. The example in Figure 6A shows immunoblotting for phospho-Akt/Akt after a 24hr exposure of lenses in organ culture to either the pan-PI3K inhibitor LY294002, of which Akt is a downstream target, or the Akt-specific inhibitor MK-2206. Immunoblot studies are also an effective way of determining how blocking specific signaling pathways or their effector molecules with small molecule inhibitors impacts cellular processes. In Figure 6A, the immunoblot for p-p70S6K/p70S6K is used to show that blocking PI3K/Akt signaling induces autophagy signaling. Immunoblotting for LAMP-1, expressed on lysosomes that fuse with autophagosomes to form the autophagolysosomes that digest organelles being eliminated from cells, provides additional evidence for the induction of an autophagy mechanism. In addition, immunoblot analysis is a highly effective approach to demonstrate the impact of signaling pathway inhibitors on the elimination of lens organelles. In Figure 6B an example is provided that shows that inhibition of the PI3K/Akt signaling axis in lens organ culture leads to removal of non-nuclear organelles including mitochondria (using an antibody to the mitochondria membrane protein TOM20) and endoplasmic reticulum (using an antibody to the ER resident protein calreticulin).

### Use of immunolocalization analysis to examine outcomes of the exposure of lenses in organ culture to signaling pathway inhibitors on the induction of autophagy and the premature elimination of lens organelles

**Figure 7:**
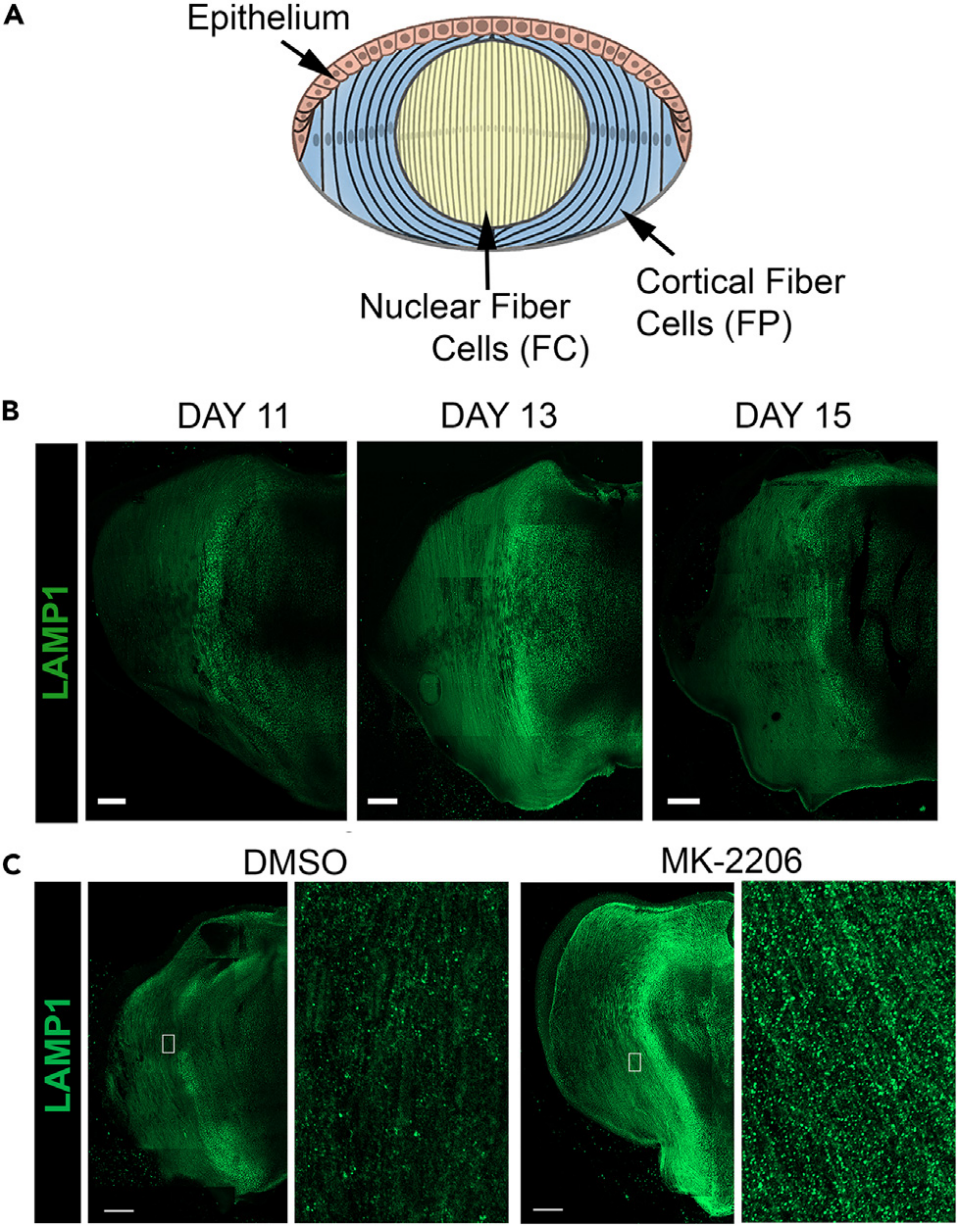
Expression of the lysosomal marker LAMP1 in lens fiber cells (A) Model illustrating different differentiation-state specific regions of the lens. (B) LAMP1 shows lysosomes accumulate in the fiber cell zone at the time of organelle removal during lens development. Scale bar, 100μm. (C) Inhibition of the PI3K/Akt signaling axis with the Akt inhibitor MK-2206 results in premature induction of lysosomes in lens fiber cells. Scale bar, 100μm.

An advantage of studying developmental processes using the chick embryo lens model is the fact that a large spectrum of lens cell differentiation stages is present at any stage of development, from undifferentiated lens epithelial cells through each phase of lens fiber cell differentiation, as modeled in Figure 7A. Immunolocalization of lens sections with antibodies to signaling pathways, autophagic processes, and specific lens organelles makes it possible to examine differentiation-state specific expression and/or activation during development and the outcome/impact of the exposure of lenses in culture to signaling pathway inhibitors. As an example, Figure 7B shows confocal imaging of LAMP-1 immunolabeling revealing that the differentiation-state specific expression of lysosomal vesicles, a component of the normal autophagy mechanism, changes with lens development. Figure 7C shows the premature induction of this autophagy pathway when lenses are exposed to an inhibitor of the PI3K/Akt signaling axis in organ culture.

**Figure 8:**
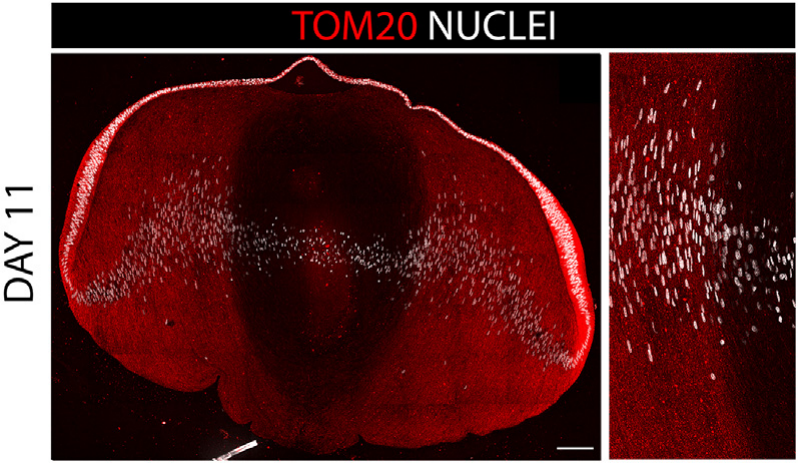
Detection of mitochondria using antibody to TOM20 Cryosection of an E11 chick embryo lens immunolabeled for the mitochondrial protein TOM20 and co-labeled with the nuclear marker DAPI. Confocal image across the entire lens (left panel). Zoomed in view at the border between the cortical and nuclear fiber cells (right panel). Scale bar, 100μm.

Immunolocalization is also an effective approach to examine the presence of lens organelles during lens development and the impact of blocking various signaling pathways on the premature elimination or, conversely, preservation, of lens organelles. An example of immunolabeling of lens mitochondria in an embryonic day 11 chick lens is shown in Figure 8. The confocal images provided show a view of the entire lens at both low (Figure 8, left panel) and high (Figure 8, right panel) magnifications.

## Quantification and statistical analysis

For immunolocalization studies images are acquired with a Zeiss confocal microscope (LSM800) and analyzed using Zeiss’ Zen software. Quantification was performed with Zen’s line scan intensity software, using the profile tool to compare fluorescence intensity across specific regions of lens fiber cells and under the different treatment conditions.

For the immunoblot studies performed by traditional Western blot, the membranes are imaged using a FluorChem F&M Imager and quantified using Digital Darkroom and AlphaView SA. For immunoblot studies performed using a WES machine, Compass software was used to analyze the data. Unpaired t-tests were used for statistical analysis.

## Limitations

The developmental timing of chick embryos varies depending on the vendor and what time of day they begin incubation of the embryonated eggs and needs to be determined before beginning any developmental analysis. When performing studies in the lens organ culture that are focused on lens fiber cells, it is important to perform dose response and timing of exposure studies. It is not recommended to rely only on published values for concentrations that were determined in monolayer cultures, as the penetrance to the center of the lens must be kept in mind. The efficacy of an inhibitor concentration on the lens fiber cell population should be determined to validate approaches. To become proficient with the lens microdissection procedure requires practice to assure accuracy of separation of the different region of differentiation and reproducibility of results. This can be tested by running immunoblots for proteins that are specific to each region of differentiation.

## Troubleshooting

### Problem 1

While embedding/freezing lenses, the lenses can become incorrectly oriented in the tissue freezing medium (Immunolocalization protocol – Step 11. Freezing). This will result in cryosections that are cut at angles that impair analysis.

### Potential solution

Check that the equatorial epithelium is facing up and use sufficient levels of the ethanol/dry ice bath so that the lens freezes as quickly as possible.

### Problem 2

Weak signals in either the immunolocalization (Immunolocalization protocol – Step 13. Immunostaining) or immunoblot protocols (Immunoblot protocol – Step 14. WES immunoblot/Step 15. Western blot).

### Potential solution

Incubation periods and antibody dilutions may need to be adjusted for optimal results.

### Problem 3

No signal when performing WES immunoblot studies (Immunoblot protocol – Step 14. WES immunoblot).

### Potential solution

While many antibodies have been validated for use with the WES, occasionally an antibody does not give a good signal with this technology. First, the antibody efficacy should be confirmed using a positive control known to express the protein of interest. If that fails, it is advised that the studies be performed instead using traditional Western blot protocol.

### Problem 4

Lens organ cultures develop opacities (Preparing lens organ cultures – Step 4. Lens organ cultures).

### Potential solution

Practice making the lens organ cultures to develop better handling and avoid injuring the lens. Take extra care not to grab the lenses too hard with the forceps when removing them from the vitreous.

### Problem 5

Immunostaining is not labeling the protein of interest (Immunolocalization protocol – Step 13. Immunostaining).

### Potential solution

Certain antibodies require staining using an antigen retrieval protocol in which samples are boiled in sodium citrate buffer (prior to incubating with the antibody) to make antigenic sites more accessible.

## Resource availability

### Lead contact

Dr. A. Sue Menko (sue.menko@jefferson.edu).

### Materials availability

This study did not generate new unique reagents.

## Data Availability

The published article includes all datasets generated or analyzed during this study.
